# Prostaglandin E Receptor Subtype 4 Signaling in the Heart: Role in Ischemia/Reperfusion Injury and Cardiac Hypertrophy

**DOI:** 10.1155/2016/1324347

**Published:** 2016-04-13

**Authors:** Lei Pang, Yin Cai, Eva Hoi Ching Tang, Michael G. Irwin, Haichun Ma, Zhengyuan Xia

**Affiliations:** ^1^Department of Anesthesiology, The First Hospital, Jilin University, Jilin 130021, China; ^2^Department of Anesthesiology, The University of Hong Kong, Pokfulam, Hong Kong; ^3^Department of Pharmacology and Pharmacy and State Key Laboratory of Pharmaceutical Biotechnology, The University of Hong Kong, Pokfulam, Hong Kong; ^4^School of Biomedical Sciences, The University of Hong Kong, Pokfulam, Hong Kong

## Abstract

Prostaglandin E_2_ (PGE_2_) is an endogenous lipid mediator, produced from the metabolism of arachidonic acids, upon the sequential actions of phospholipase A2, cyclooxygenases, and prostaglandin E synthases. The various biological functions governed by PGE_2_ are mediated through its four distinct prostaglandin E receptors (EPs), designated as EP1, EP2, EP3, and EP4, among which the EP4 receptor is the one most widely distributed in the heart. The availability of global or cardiac-specific EP4 knockout mice and the development of selective EP4 agonists/antagonists have provided substantial evidence to support the role of EP4 receptor in the heart. However, like any good drama, activation of PGE_2_-EP4 signaling exerts both protective and detrimental effects in the ischemic heart disease. Thus, the primary object of this review is to provide a comprehensive overview of the current progress of the PGE_2_-EP4 signaling in ischemic heart diseases, including cardiac hypertrophy and myocardial ischemia/reperfusion injury. A better understanding of PGE_2_-EP4 signaling should promote the development of more effective therapeutic approaches to treat the ischemic heart diseases without triggering unwanted side effects.

## 1. Introduction

Ischemia of the heart resulting from the shortage of oxygen supply can lead to the occurrence of myocardial ischemia/reperfusion injury (MI/R), presenting as a leading cause of mortality and morbidity especially in those with preexisting myocardial diseases such as cardiac hypertrophy both in developed and in developing countries [1]. In the general population, the incidence of ischemic heart diseases increases when a person becomes overweight or obese [2, 3], which could be attributed to the alterations in the control of coronary blood flow [4] or the dysregulation of adipocyte-derived hormones (such as adiponectin, leptin) in the obesity [5, 6]. The population of obesity has skyrocketed worldwide over the last three decades [7]. In addition, overweight and obesity were estimated to have caused 3.4 million deaths in 2010, most of which were from cardiovascular causes [8]. Therefore, examining ways to protect the ischemic heart and its associated diseases (like obesity, metabolic syndrome) will be of great clinical value in this industrialized world.

Prostaglandin E_2_ (PGE_2_) is an endogenous lipid mediator, which belongs to the family of eicosanoids [9]. Upon the action of phospholipase A2, arachidonic acid, the precursor of prostaglandins, is generated from phospholipids in the cell membrane [10]. Arachidonic acid is then metabolized into prostaglandin H2 by cyclooxygenase (COX) enzymes. Prostaglandin H2 is the first intermediate in the biosynthesis of prostaglandins, which requires the action of specific prostaglandin synthases. The specific synthases involved in the formation of PGE_2_ are microsomal prostaglandin E synthases- (mPGES-) 1 and mPGES-2 and cytosolic PGE_2_ synthases (cPGES) [9, 11]. PGE_2_ exerts its diverse effects by activating four subtypes of prostaglandin E receptors (EPs), designated as EP1, EP2, EP3, and EP4 [12]. Of those, the EP4 receptor is the most widely distributed subtype which exists in almost all tissues, such as the heart, adipose tissue, skeletal muscle, and lung [13–15], and is involved in various pathophysiological processes [16–18]. In particular, mice lacking EP4 exhibited slower weight gain and reduced adiposity upon high fat diet challenge when compared with wild type mice [16]. However, the lean phenotype of EP4 knockout mice is not a beneficial factor. In fact, EP4 knockout mice had a shorter life span than did the wild type mice [16]. In addition, deficiency of EP4 in mice manifests disrupted lipid metabolism due to impaired triglyceride clearance, suggesting a new dimension role of EP4 signaling in controlling lipid homeostasis [16]. Activation of PGE_2_-EP4 signaling also can exert multiple biochemical effects on the heart, suggesting the potential wide-ranging use of EP4 in both cardiovascular and metabolic disorders. However, due to the limited reports of EP4 in ischemic heart under complicated disease states, in this review, we thus summarize the current progress regarding the role of the PGE_2_-EP4 signaling in ischemic heart diseases, including cardiac hypertrophy and MI/R, which has been obtained from studies using genetic knockout mouse and pharmacological interventions.

## 2. Prostaglandin E Receptor Subtype 4: Structure and Signaling

As one of the seven-transmembrane G-protein-coupled receptors, EP4 (originally misidentified as EP2 subtype [19]) shares the structure properties of G-protein-coupled receptors. It has an extracellular N-terminus, a seven-transmembrane domain connected by three extracellular loops and three intracellular loops, and an intracellular C-terminus [20]. The N-glycosylation sites in the second extracellular loop of EP4 are important for the ligand binding [21]. EP4 has the longest intracellular C-terminus and third intracellular loop out of the four EP receptors. The human EP4 receptor is comprised of 488 amino acid residues, while the murine EP4 receptor has two isoform variants that consist of 488 and 513 amino acid residues, respectively [22, 23]. The sequence homology of EP4 between these two species reaches up to 88% [21].

**Figure 1:**
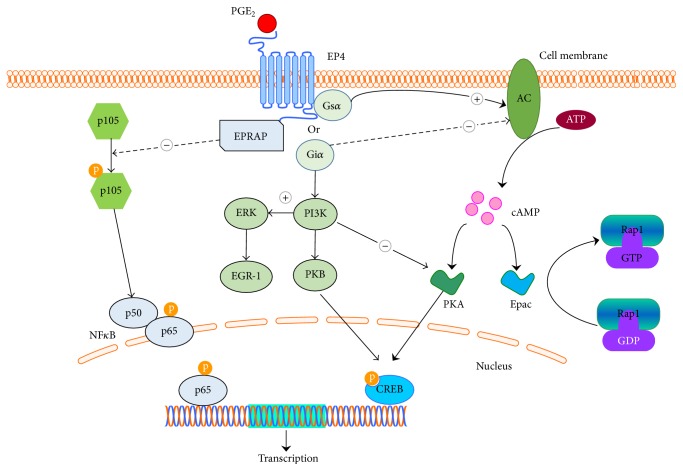
Differential signaling pathway of EP4. In response to PGE_2_, activation of EP4 stimulates stimulatory G*α* protein (Gs*α*)/cyclic adenosine monophosphate (cAMP)/protein kinase A (PKA)/cAMP response element binding protein (CREB) pathway or Gs*α*/cAMP/exchange protein directly activated by cAMP (Epac) pathway. EP4 is also coupled to inhibitory G*α* protein (Gi*α*), which inhibits the cAMP/PKA/CREB pathway. Furthermore, EP4 activates phosphatidylinositol 3-kinase (PI3K) through activation of Gi*α*. Activation of PI3K not only stimulates the protein kinase B (PKB)/CREB pathway but also induces the expression of early growth response factor 1 (EGR-1) through extracellular signal-regulated kinase signaling. Prostaglandin E receptor 4-associated protein (EPRAP) can inhibit phosphorylation of p105 and further suppress the activation of nuclear factor kappa B (NF*κ*B). AC = adenylyl cyclase; ATP = adenosine triphosphate; GDP = guanosine diphosphate; GTP = guanosine triphosphate.

The downstream effectors of G-protein-coupled receptors are G-proteins, which consist of *α*, *β*, and *γ* subunits. Upon ligand binding, the conformational change in G-protein-coupled receptors triggers the dissociation of G*α* from the G*βγ* subunits [22]. EP4 is coupled to stimulated G*α* (Gs*α*), which leads to the production of cyclic adenosine monophosphate (cAMP) in response to PGE_2_ [23]. The increased intracellular cAMP level subsequently activates its major target protein kinase A (PKA). PKA then phosphorylates downstream protein, cAMP response element binding protein (CREBP), which is a nuclear transcriptional factor [24]. The activated CREBP then binds to specific sites and regulates the expression of certain genes, such as B-cell lymphoma 2 and tumor necrosis factor *α* (TNF*α*), which are involved in development of ischemic heart disease [25]. In addition to PKA, another downstream molecule of Gs*α*/cAMP is exchange protein directly activated by cAMP (Epac). Epac consists of Epac1 and Epac2. Both Epac isoforms can convert their downstream protein Ras-related protein 1 from inactivated guanosine diphosphate form to activated guanosine triphosphate form, which leads to the initiation of downstream signaling cascades [26]. EP4 is also coupled to the inhibitory G*α* (Gi*α*) [27]. In response to PGE_2_, Gi*α* inhibits adenylyl cyclase activity leading to the reduced production of cAMP [28]. Furthermore, through activation of Gi*α*, EP4 mediates phosphatidylinositol 3-kinase- (PI3K-) dependent pathway [29]. Activation of PI3K inhibits PKA activity but activates protein kinase B, which also can phosphorylate CREBP [30]. On the other hand, EP4 induces the expression of early growth response factor 1 through the PI3K/extracellular signal-regulated kinase (ERK) signaling pathway, which leads to the expression of PGE_2 _synthase, suggesting a positive feedback loop between EP4 and PGE_2 _production [23, 31]. Furthermore, yeast two-hybrid screening of human bone marrow complementary DNA with EP4 protein reveals that a protein named prostaglandin E receptor 4-associated protein (EPRAP) binds to the intracellular C-terminus of EP4 [32]. The interaction of EPRAP and EP4 inhibits stimulus-induced NF*κ*B p105 phosphorylation and thus suppresses activation of NF*κ*B [33] (Figure 1).

Besides EP4 receptor, there are another three GPCRs, EP1, EP2, and EP3, which depend on G-protein to transduce downstream signals and mediate PGE_2_ actions [12]. EP1 receptor couples to Gq*α* protein and thus induces phospholipase C/inositol-1,4,5-trisphosphate signaling and leads to intracellular calcium mobilization [34]. The same as EP4 receptor, EP2 couples to Gs*α* protein and activates adenylate cyclase to produce cAMP, while EP3 is associated with Gi*α* and inhibits cAMP production [35]. These EP subtypes have their unique expression patterns and associate to distinct downstream G-proteins and thereby lead to PGE_2_ being the most versatile prostanoid.

## 3. Myocardial Ischemia/Reperfusion Injury

Myocardial ischemia caused by partial or complete occlusion of coronary arteries and the subsequent recovery of blood flow induced additional cardiac damage (ischemia/reperfusion injury) are leading causes for death around the world [36]. The underlying pathophysiology of myocardial I/R injury likely involves many factors, such as reactive oxygen species formation [37], altered cardiac energy metabolism [38], activation of cell apoptosis [39], and inflammatory responses [40].

During cardiac ischemia, the PGE_2_ level is significantly increased [41], and this increase may be a consequence of hypoxia inducible factor- (HIF-) 1*α*/COX-2/PGE_2_ pathway activation. Under ischemia conditions, the HIF-1*α* level starts to accumulate in the nucleus, be heterodimerized with *β* subunit, and initiate the transcription of genes which are involved in cell survival, angiogenesis, apoptosis, vascular remodeling, and glucose metabolism [42]. COX-2 has been proved to be a downstream protein of HIF-1*α* in various cell types, such as carcinoma cell lines HT29 [43] and human bronchial epithelial BEAS-2B cells [44]. COX-2 has been reported to be induced in the heart during I/R [45] and its expression was positively associated with the expression of HIF-1*α* at the site of recent acute myocardial infarction [46], despite the fact that direct evidence regarding whether or not COX-2 was transcriptionally controlled by HIF-1*α* in cardiomyocytes is lacking. Thus, as the major metabolite of COX-2, cardiac PGE_2_ may be produced through HIF-1*α*/COX-2 axis during cardiac ischemia.

The increased PGE_2_ level may play a beneficial role during cardiac I/R through EP4 receptor [15, 47]. Indeed, endogenous PGE_2_ protects the heart from I/R injury* in vitro* and* in vivo* [15]. In isolated perfused working hearts, there was a greater degree of functional damage to the myocardium (e.g., decreased developed tension, increased diastolic tension and creatine kinase (marker of myocardial injury) release) in EP4 knockout hearts after global ischemia when compared with wild type hearts [15]. In accordance with this result, mice lacking EP4 developed a greater degree of myocardial infarction size following I/R injury when compared with wild type mice* in vivo* [15]. Likewise, pharmacological intervention with an EP4 agonist significantly reduced infarct size and improved cardiac function, including left ventricular contraction and dilatation when compared with vehicle treated animals [47]. In line with this, the ischemic preconditioning-induced cardioprotection is completely lost in HIF-1*α* ^+/−^ mice [48]. Deficiency of hypoxia inducible transcription factor-prolyl hydroxylase domain-1 (PHD-1) in the mice significantly attenuated MI/R injury through reduced apoptosis by induction of HIF-1*α* [49], and COX-2 serves a protective role against myocardial I/R injury [50, 51]. These data provide additional evidence that PGE_2_-EP4 signaling sourced from HIF-1*α*/COX-2 axis is cardioprotective in the ischemic heart.

The subsequent downstream signaling of EP4 during the myocardial I/R injury has not been well documented yet. However, there are multiple potential downstream pathways. (1) cAMP-PKA pathway: Through the activation of EP4 receptor on the cell membrane, adenylyl cyclase catalyzes the conversion of adenosine triphosphate to cAMP. There are at least 9 isoforms of adenylyl cyclase; among them, adenylyl cyclase V and adenylyl cyclase VI are mainly expressed in the mammalian myocardium [52]. In the mice with cardiac adenylyl cyclase VI overexpression, adverse left ventricular remodeling was attenuated with preserved left ventricular contractile function and reduced mortality after myocardial ischemia [53]. In addition, through activating the downstream protein cAMP, PKA presents a cardioprotective role after ischemia possibly through its negative-inotropic effect during sustained ischemia [54]. In this study, the negative-inotropic effects of postischemic effluent can be significantly suppressed by preincubation with EP2 antagonist (AH6809) or EP4 antagonist (AH23848), indicating a protective role of both EP2 and EP4 receptor during I/R injury. However, unlike EP4 receptor, there is still no direct evidence showing that EP2 receptor can mediate the cardioprotective role of PGE_2_ in the postischemic heart during reperfusion* in vivo* and this warrants further investigation. These data suggested that cAMP-PKA may be responsible for the PGE_2_-EP4-mediated cardioprotection during myocardial ischemia. (2) Stat3 signaling: In addition to its role in the cardiac hypertrophy, Stat3 signaling activation also plays a causal role in ischemic postconditioning mediated cardioprotection [5]. Multiple lines of evidence suggested that activation of Stat3 signaling exerts cardioprotective effects during I/R injury through scavenging of reactive oxygen species [55] or improving mitochondrial function [56]. In cardiomyocytes, PGE_2_ activated Stat3 signaling through EP4 receptor in a concentration- and time-dependent manner [57]. Moreover, MI/R-induced tissue injury involves activation of cell apoptosis [39]. PGE_2_ is reported to prevent myocardial apoptosis through activation of Stat3 and ERK1/2 in doxorubicin-induced apoptosis model in neonatal rat ventricular cardiomyocytes [58], suggesting that activation of Stat3 signaling may contribute to PGE_2_-EP4-mediated cardioprotective role during I/R injury. In addition, despite the fact that there is no direct evidence showing that activation of EP4 receptor exerts antiapoptotic role in cardiomyocytes during I/R injury, it has been proved that PGE_2_ protects normal and transformed intestinal epithelial cells from diverse stimuli-induced apoptosis* via* EP4 receptor [59]. Consistently, another study showed that EP4 agonist (L-902688) significantly inhibits the cell death during* in vivo* focal cerebral ischemia [60]. Taken together, these data suggested that PGE_2_-EP4 signaling may mediate cardioprotection through attenuating cell apoptosis during myocardial ischemia.

**Figure 2:**
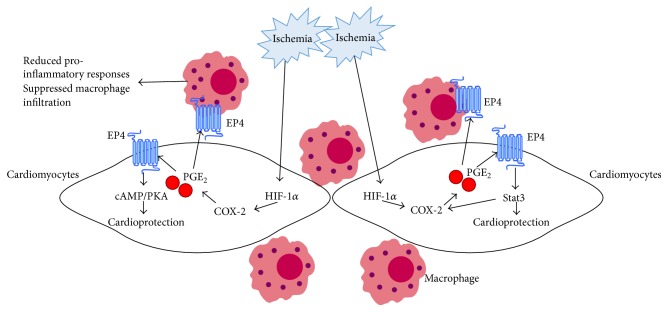
Schematic representation of the proposed role of EP4-mediated signaling under myocardial ischemia. During cardiac ischemia, increased PGE_2_ level may be a consequence of increased hypoxia inducible factor- (HIF-) 1*α*/cyclooxygenase-2 (COX-2) activation. PGE_2_ may play a beneficial role in cardiac ischemia/reperfusion injury through EP4/cyclic adenosine monophosphate (cAMP)/protein kinase A (PKA) or EP4/signal transducer and activator of transcription 3 (Stat3) signaling pathway. In addition, synthesized PGE_2_ in cardiomyocytes also diffuses into adjacent infiltrated macrophages or other inflammatory cells, protects the heart from ischemia/reperfusion injury through binding with EP4 receptor, and exerts anti-inflammatory effects.

During ischemia, inflammatory cells like macrophages may migrate into the ischemic myocardium and produce proinflammatory cytokines and chemokines, including TNF*α*, interleukin 6 (IL6), IL-1*β*, and monocyte chemotactic protein 1 (MCP-1), which further exacerbate myocardial I/R injury [40]. Thus, it is possible that the anti-inflammatory effect of EP4 on macrophages may contribute to the beneficial role of EP4 in cardiac ischemia. Indeed, EP4 agonist significantly attenuated the production of TNF*α*, IL-6, IL-1*β*, and MCP-1 as well as macrophage infiltration in the heart after ischemia [47]. Of note, the EP4-Stat3 signaling may establish a positive feedback loop through controlling the expression of COX-2 in cardiomyocytes, while the newly secreted PGE_2_ may not only trigger greater Stat3 activation but also exert anti-inflammatory effect on infiltrated inflammatory cells in a paracrine way (Figure 2). Thus, EP4 receptor may confer protection against I/R injury at multiple levels. EP4 agonist may provide a novel approach to limit the damage from myocardial I/R injury.

## 4. Cardiac Hypertrophy

Pathological cardiac hypertrophy is a slow adaptive response to various types of extracellular stressors, including increased hemodynamic load [61], neurohormones [62], growth factors [63], and cytokines [64]. At the early stage, cardiac hypertrophy is compensatory to maintain the circulatory system homeostasis. However, the severe and sustained workload on the heart may trigger the cardiac remodeling process and increase the risk of cardiac dysfunction and, ultimately, the development of heart failure [65] and the underlying mechanism is incompletely understood. A better understanding of the signal transmission from cell surface to the nuclear transcription activities in response to various hypertrophic stimuli may yield novel therapeutic approaches to treat cardiac hypertrophy.

In ventricular myocytes, PGE_2_ significantly increased total protein synthesis (measured by [^3^H]-phenylalanine uptake), cell surface area, and hypertrophic maker genes, including atrial natriuretic peptide (ANP) and brain natriuretic peptide (BNP), in a dose dependent manner [66–68]. These hypertrophic effects of PGE_2_ were conserved* in vivo*. Using a mouse model of myocardial infarction, injection with NS-398 or rofecoxib (COX-2 selective inhibitors) in mice significantly downregulated cardiac PGE_2_ production and reduced cardiac hypertrophy as determined by myocyte cross-sectional area when compared with vehicle treated mice [69]. Similarly, mice with global knockout of mPGES-1, which is in charge of the inducible PGE_2_ synthase, also exhibited decreased cardiac PGE_2_ levels, myocyte cross-sectional area, and cardiomyocyte surface area after myocardial infarction when compared to wild type mice, suggesting that PGE_2_ positively regulates cardiac hypertrophy* in vitro* and* in vivo* [70].

Activation of EP4 receptor signaling contributes to the PGE_2_-mediated cardiac hypertrophy, since EP4 specific antagonist (L-161982 or ONO-AE3-208) significantly blocked the hypertrophic actions of PGE_2_, including the protein synthesis, mRNA expression of ANP and BNP in neonatal cardiac cells [66, 68]. In accordance with these observations, myocyte cross-sectional area was significantly smaller in cardiac-specific EP4 knockout mice after myocardial infarction when compared with wild type mice, suggesting that the lack of EP4 receptor signaling in cardiomyocytes alleviated cardiac hypertrophy after myocardial infarction [71]. By contrast, global EP4 knockout mice did not affect the myocyte cross-sectional area and heart/body weight ratio under basal conditions [15] or pressure overload-induced cardiac hypertrophy through transverse aortic constriction (TAC) treatment [72] as compared with wild type mice. There are several possible explanations regarding the discrepancy among these studies. (1) The development of cardiac hypertrophy is a very slow process, and thus if there are no continuous hypertrophic stimuli, it may take a long time to develop cardiac hypertrophy. Therefore, it may be difficult to detect significant difference in the development of cardiac hypertrophy between EP4 wild type and knockout mice under basal conditions. (2) The wide distribution of EP4 receptor in different kinds of cells indicates its diverse biological functions* in vivo*, such as anti-inflammatory response [73] and energy metabolism [16]. The affected anti-inflammatory pathway or lipid metabolism in global EP4 knockout mice may influence the development of cardiac hypertrophy indirectly. Indeed, inflammatory process and energy metabolism are closely associated with the pathogenesis of cardiac hypertrophy [74, 75]. Thus, under basal condition or TAC-induced cardiac hypertrophy, global knockout of EP4 in mice may affect the pathogenesis of cardiac hypertrophy through other compensatory pathways. (3) Different cardiac hypertrophic stimuli activate diverse signaling cascades and may have distinct cardiomyocyte gene expression pattern [65]. It is possible that PGE_2_-EP4 signaling is only involved in myocardial infarction—but not pressure overload-mediated cardiac hypertrophy. (4) In the study of Hara et al. [72], the heart/body weight ratios were only compared between EP4 wild type and knockout mice after 4 weeks of TAC treatment, a time point at which generally the pressure overload-induced cardiac hypertrophy has reached the maximal level. Thus, comparison should be performed at both the early and late phase of the disease to get a solid conclusion. Through these explanations, it is tempting to speculate that EP4 in cardiomyocytes may be the endogenous ligand to mediate the hypertrophic effects of PGE_2_ both* in vitro* and* in vivo*, but its definite role in this pathology needs to be confirmed in future studies.

The additional challenge is to identify the subsequent downstream molecules in EP4-mediated cardiac hypertrophy. Mammalian target of rapamycin (mTOR) has emerged as an important regulator of cardiac hypertrophy [76]. There is study that reported that PGE_2_ activates the mTOR complex 1 pathway through an EP4/cAMP/PKA mediated mechanism in the human pancreatic carcinoma cell line PANC-1 [77]. Besides PKA, cAMP also exerts its biological functions through Epac. Despite the fact that cAMP-producing *β*-adrenergic stimulation induces cardiac hypertrophy [78], cAMP/PKA or cAMP/Epac signaling is not likely to mediate the hypertrophic effects of PGE_2_ in cardiomyocytes, since all the treatment like cAMP activator (forskolin), cAMP inhibitor (SQ-22536), PKA inhibitor (H89), and Epac activator (8-CPT-2Me-cAMP) at different concentrations had no effect on PGE_2_-induced protein synthesis in ventricular myocytes [57, 68, 79]. Thus, mTOR may not be able to mediate the hypertrophic effects of PGE_2_/EP4 in cardiomyocytes.

It is well known that mitogen-activated protein kinases (MAPKs) signaling cascade is a prominent player involved in the hypertrophic reaction with its four best characterized subfamilies, extracellular signal-regulated kinases (ERK1/2 or P42/P44 MAPK), c-Jun N-terminal kinase (JNK), p38 MAPK, and ERK5 [80]. The ERK1/2 inhibitor, U0126, significantly reduced the hypertrophic effect of PGE_2_ in ventricular myocytes, whereas the p38 MAPK blocker (SB203580) and JNK inhibitor (SP600125) have no such effect [68]. In addition, activation of ERK1/2 by PGE_2_ was strongly suppressed by EP4 antagonist ONO-208 compound, indicating that ERK1/2 signaling cascade was involved in PGE_2_-EP4-mediated cardiac hypertrophy [57, 68]. However, the involvement of ERK5 on PGE_2_-induced protein synthesis remains undefined.

Activation of G-protein-coupled receptors has been shown to transactivate epidermal growth factor receptors (EGFR), which can result in the activation of ERK1/2 signaling [81, 82]. Indeed, EP4 antagonist L-161982 significantly inhibited the phosphorylation of EGFR by PGE_2_ in ventricular myocytes. Furthermore, EGFR inhibitor AG-1478 totally blocked activation of ERK1/2 by PGE_2_ and PGE_2_-EP4-mediated protein synthesis, suggesting that activation of EGFR bridges PGE_2_/EP4 and ERK1/2 signaling and also is responsible for PGE_2_-induced protein synthesis in cardiomyocytes [79].

**Figure 3:**
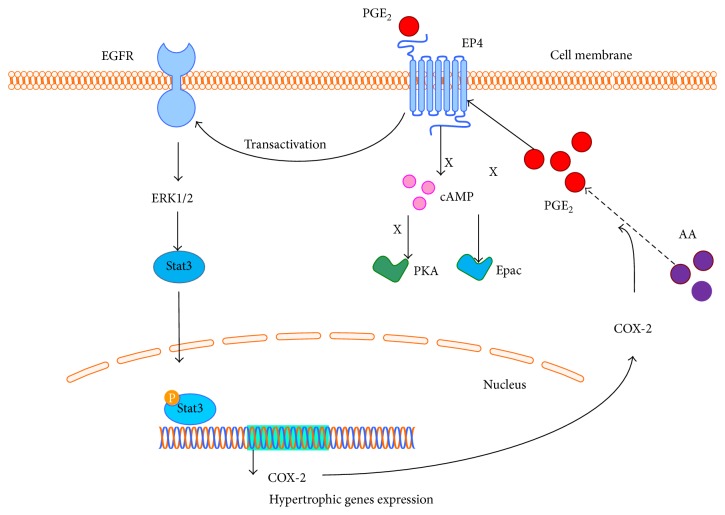
Simplified scheme of the EP4 signaling in PGE_2_-mediated hypertrophic actions. In response to PGE_2_, activation of EP4 transactivates epidermal growth factor receptors (EGFR), which can result in the activation of extracellular signal-regulated kinases (ERK1/2). ERK1/2 in turn activated signal transducer and activator of transcription 3 (Stat3) pathway. Once Stat3 signaling is activated in the nucleus, the hypertrophic genes will be expressed to mediate the protein synthesis. In addition, cyclooxygenase-2 (COX-2) expression is also stimulated by Stat3 signaling, which will further metabolize the formation of PGE_2_ from arachidonic acid (AA) and trigger the greater activation of Stat3 signaling in a positive feedback loop. Cyclic adenosine monophosphate (cAMP)/protein kinase A (PKA) or cAMP/exchange protein directly activated by cAMP (Epac) pathways are not involved in the PGE_2_-EP4-mediated hypertrophic actions in ventricular myocytes.

As reported in several studies, signal transducer and activator of transcription 3 (Stat3) pathway is another signaling cascade that plays a major role in the development of cardiac hypertrophy. Multiple* in vitro* and* in vivo* studies suggested that activation of Stat3 promotes cardiomyocyte hypertrophy in response to various stimuli through transcriptional control of hypertrophic genes expression [83, 84]. PGE_2_ induced Stat3 activation in cardiomyocytes in a concentration- and time-dependent manner, while ERK1/2 inhibitor (U0126) and EP4 antagonists (GW627368X or AH23848B) significantly suppressed PGE_2_-induced Stat3 activation [57]. In Stat3-silenced cardiomyocytes, the PGE_2_-mediated protein synthesis was dramatically inhibited [57], suggesting that activation of Stat3 works downstream of PGE_2_-EP4-ERK1/2-mediated cardiac hypertrophy* in vitro*. Accordingly, myocardial infarction induced cardiac hypertrophy was accompanied with significantly increased Stat-3 phosphorylation in wild type controls, but the increase of Stat-3 phosphorylation was absent in the heart from cardiomyocyte-specific EP4 knockout mouse, suggesting that EP4-Stat3 signaling is conserved* in vivo* and responsible for cardiac hypertrophy [71]. Furthermore, once Stat3 signaling is activated in the nucleus, the gene expression of COX-2 will be stimulated, which will metabolize the first step in the formation of PGE_2_ from arachidonic acid and trigger the greater activation of Stat3 signaling in a positive feedback loop [85]. Thus, these findings together demonstrated the pathophysiological importance of PGE_2_ signaling through EP4-EGFR-ERK1/2-Stat3 cascade that was involved in the development of cardiac hypertrophy (Figure 3).

## 5. Conclusion

Cardiac hypertrophy is not only a risk factor for myocardial ischemia, but also part of the response of MI/R-induced tissue injury, which further promotes the extent of ischemia. It is very interesting that activation of PGE_2_-EP4 signaling may impact on ischemic heart events through totally opposite pathways. EP4 may mediate the cardiac hypertrophy through EGFR/ERK1/2/Stat3 signaling, whereas PGE_2_-EP4 signaling may protect the heart from MI/R injury through cAMP/PKA or Stat3 pathway. Despite the fact that there is still no direct evidence showing the role of Stat3 signaling in EP4-mediated cardioprotection, the* in vivo* model, cardiac-specific EP4 knockout mice with myocardial infarction, exhibited decreased hypertrophic changes but worsened cardiac function, suggesting that activation of EP4 signaling may contribute to the compensatory survival of cardiomyocytes for maintaining the normal cardiac function. In the future, with regard to the potential detrimental effects of EP4, special caution is needed when evaluating how EP4 can be preferably activated in the heart without triggering unwanted side effects. In addition, the role of PGE_2_-EP4 signaling pathway in the heart should be further explored in detail, with the aim of developing therapeutic approaches to treat the patients with ischemic heart disease and its associated diseases.
